# Correction to ‘Global Hfq-mediated RNA interactome of nitrogen starved *Escherichia coli* uncovers a conserved post-transcriptional regulatory axis required for optimal growth recovery’

**DOI:** 10.1093/nar/gkae375

**Published:** 2024-05-06

This is a correction to: Josh McQuail, Gianluca Matera, Tom Gräfenhan, Thorsten Bischler, Per Haberkant, Frank Stein, Jörg Vogel, Sivaramesh Wigneshweraraj, Global Hfq-mediated RNA interactome of nitrogen starved *Escherichia coli* uncovers a conserved post-transcriptional regulatory axis required for optimal growth recovery, *Nucleic Acids Research*, Volume 52, Issue 5, 21 March 2024, Pages 2323–2339, https://doi.org/10.1093/nar/gkad1211

The authors wish to correct a spelling error in the Graphical Abstract (post-transctiptional to post-transcriptional).

The authors also wish to correct the caption of Figure 2D from:

(D) As above, showing only proteins whose mRNA interacted with SdsR, coloured by the SdsR binding site as determined though RIL-seq. Interaction regions overlapping the RBS (defined here as 40bp upstream to 15bp downstream of the AUG) are shown in green, interactions internal to the coding sequence or in the 3′ UTR are shown in red, interactions in the 5′UTR not overlapping the RBS are shown in blue, targets with multiple binding sites within the mRNA are shown with split colour and poorly defined binding sites and binding sites within intergenic regions in the same transcript are shown in grey. Schematic representation of the categories of sRNA binding site, and the expected regulatory outcome on protein levels in the wild-type and ΔsdsR bacteria are schematically shown.

To

(D) As above, showing only proteins whose mRNA interacted with SdsR, coloured by the SdsR binding site as determined though RIL-seq. Interaction regions overlapping the RBS (defined here as 40bp upstream to 15bp downstream of the AUG) are shown in **red**, interactions internal to the coding sequence or in the 3′ UTR are shown in **blue**, interactions in the 5′UTR not overlapping the RBS are shown in **green**, targets with multiple binding sites within the mRNA are shown with split colour and poorly defined binding sites and binding sites within intergenic regions in the same transcript are shown in grey. Schematic representation of the categories of sRNA binding site, and the expected regulatory outcome on protein levels in the wild-type and ΔsdsR bacteria are schematically shown.

These changes do not affect the results, discussion and conclusions presented in the article. The published article has been updated.

